# Dialectical Behavioral Therapy for Adolescents (DBT-A): a clinical Trial for Patients with suicidal and self-injurious Behavior and Borderline Symptoms with a one-year Follow-up

**DOI:** 10.1186/1753-2000-5-3

**Published:** 2011-01-28

**Authors:** Christian Fleischhaker, Renate Böhme, Barbara Sixt, Christiane Brück, Csilla Schneider, Eberhard Schulz

**Affiliations:** 1 Division of Child and Adolescent Psychiatry and Psychotherapy, Department of Psychiatry and Psychosomatic Medicine, Albert Ludwig University Medical Center Freiburg, Hauptstr. 8, 79104 Freiburg, Germany; 2 Gemeinschaftspraxis Kinder- und Jugendpsychiatrie Dres. Renate Böhme und Mariele Ritter-Gekeler, Hauptstr. 49, 79379 Müllheim, Germany

## Abstract

**Background:**

To date, there are no empirically validated treatments of good quality for adolescents showing suicidality and non-suicidal self-injurious behavior. Risk factors for suicide are impulsive and non-suicidal self-injurious behavior, depression, conduct disorders and child abuse. Behind this background, we tested the main hypothesis of our study; that Dialectical Behavioral Therapy for Adolescents is an effective treatment for these patients.

**Methods:**

Dialectical Behavioral Therapy (DBT) has been developed by Marsha Linehan - especially for the outpatient treatment of chronically non-suicidal patients diagnosed with borderline personality disorder. The modified version of DBT for Adolescents (DBT-A) from Rathus & Miller has been adapted for a 16-24 week outpatient treatment in the German-speaking area by our group. The efficacy of treatment was measured by a pre-/post- comparison and a one-year follow-up with the aid of standardized instruments (SCL-90-R, CBCL, YSR, ILC, CGI).

**Results:**

In the pilot study, 12 adolescents were treated. At the beginning of therapy, 83% of patients fulfilled five or more DSM-IV criteria for borderline personality disorder. From the beginning of therapy to one year after its end, the mean value of these diagnostic criteria decreased significantly from 5.8 to 2.75. 75% of patients were kept in therapy. For the behavioral domains according to the SCL-90-R and YSR, we have found effect sizes between 0.54 and 2.14.

During treatment, non-suicidal self-injurious behavior reduced significantly. Before the start of therapy, 8 of 12 patients had attempted suicide at least once. There were neither suicidal attempts during treatment with DBT-A nor at the one-year follow-up.

**Conclusions:**

The promising results suggest that the interventions were well accepted by the patients and their families, and were associated with improvement in multiple domains including suicidality, non-suicidal self-injurious behavior, emotion dysregulation and depression from the beginning of therapy to the one-year follow-up.

## Background

Adolescents with borderline personality disorder (BPD) show many similarities to adult patients in terms of early history, current behaviors and coexisting Axis I disorders. Inpatient studies have demonstrated that BPD in adolescents can be reliably diagnosed, occurs frequently and has concurrent validity with some temporary instability [1,2]. While caution is warranted, formal assessment of BPD in adolescents may yield more accurate and effective treatment for adolescents experiencing BPD symptomatology [3].

Adolescents with BPD display recurrent suicidal behavior, gestures, threats or non-suicidal self-injury (NSSI); e. g. cutting or burning. Suicide threats and attempts are very common. Follow-up studies have found that 10 - 50% of adolescents attempting suicide make suicide attempts in the future. Out of these, up to 11% eventually die by suicide [4]. Unfortunately, up to 77% of adolescent suicide attempters either do not attend outpatient treatment or drop out before learning how to tolerate distress better and how to regulate their emotions effectively (i. e. by means of skills), without resorting to suicidal or non-suicidal self-injury [4,5].

Dialectical Behavior Therapy (DBT) has been developed by Marsha Linehan and colleagues [6] for the treatment of chronically parasuicidal adults with BPD, whereas the term parasuicide as used by Linehan included suicidal behavior. Rathus and Miller [7] have adapted DBT for suicidal adolescents with borderline personality traits for its strategies of keeping patients committed to treatment and for its focus on reducing both suicidal and quality of life interfering behaviors. Dialectical Behavior Therapy for Adolescents (DBT-A) is a manualized, 16-week behavioral treatment, that includes concurrent individual therapy once a week, family therapy as needed and a multifamily skills training group in an outpatient setting. An open clinical trial by Rathus and Miller has demonstrated the effectiveness of this DBT adaptation by means of pre-post comparisons indicating significant reduction of suicidal ideation, of general psychiatric symptoms and of borderline personality symptoms [7]. Comparing a treatment-as-usual group with a DBT-A group, Rathus and Miller have found less psychiatric hospitalizations during DBT-A treatment as well as significantly higher treatment completion rates for the DBT-A group.

Futhermore, DBT-A has been successfully implemented for an inpatient therapy setting for suicidal adolescents. DBT-A has significantly reduced behavioral incidents in comparison to treatment as usual [8].

In addition, DBT-A has been adapted for the treatment of adolescents with bipolar disorder and a promising open clinical trial has been performed by Goldstein et al. [9]. DBT-A has been adapted and modified by Fleischhaker and colleagues for use in Germany [10]. The published treatment manual was used in a pilot study at the Department of Child and Adolescent Psychiatry in Freiburg [11]. This open clinical trial validated the effectiveness of DBT-A by showing significant reduction of parasuicidal acts four weeks after the end of treatment and a drop-out rate as little as 25%. In addition, patients experienced significant improvement in global psychopathology and psychosocial adaptation. In this paper, a one-year follow-up investigation of these patients is presented

## Methods

Participation in our pilot study on DBT-A was proposed to all families with adolescent females exhibiting non-suicidal self-injurious and suicidal behavior. In order to guarantee a greater homogeneity of the sample, the pilot study was limited to female patients. For pragmatic reasons, the inclusion and exclusion criteria were defined as follows:

### Inclusion criteria

- Age at the beginning of therapy between 13 and 19 years

- Non-suicidal self-injurious and/or suicidal behavior in the past 16 weeks

- Diagnosis of BPD or existence of at least three DSM-IV criteria (Diagnostic and Statistical Manual of Mental Disorders, fourth edition) for BPD. The diagnosis of BPD was made by means of a semi-structured interview (SKID-II)

### Exclusion criteria

- Cognitive performance according to an intelligence quotient (Culture Fair Test 20; [CFT 20] [12] or HAWIK [Hamburg-Wechsler Intelligence Test for Children]) below 70

- Present psychotic disorder

- Present severe depressive episode or mania with indication for inpatient therapy

- Substance abuse or eating disorder as primary diagnosis

- Illiteracy

These inclusion and exclusion criteria correspond to those of the pilot study for DBT-A conducted by Rathus and Miller [7] in order to guarantee good comparability. In Germany, patients suffering from severe depression episodes or mania are treated in inpatient settings. Therefore, these diagnoses were added to the exclusion criteria as well.

DBT-A was carried out at our Child and Adolescent Psychiatric Outpatient Department in an outpatient setting over a period of 16 to 24 weeks. The duration of treatment varied due to school holidays. In school holidays, no multi family skills training groups were held. The adolescents kept two appointments per week: Individual therapy (one hour) and participation in the multi family skills training group (two hours). The following skills were taught in this group: Mindfulness Skills, Interpersonal Effectiveness Skills, Distress Tolerance Skills, Emotion Regulation Skills, Family Skills and "Walking the Middle Path". In the multi family skills training groups, we included up to 12 persons (up to five adolescents plus one of the parents and two therapists). In addition, we arranged regular phone contacts between individual therapist and patient as needed in order to support generalization of recently acquired skills in everyday life.

### Measures

Prior to admission to the pilot study, we implemented the following standard instruments during a diagnostic appointment:

- SKID-I (Structured Clinical Interview for DSM-IV, German version, [13])

- SKID-II (Structured Clinical Interview for DSM-IV, German version, [13])

- Parts of Kiddie-SADS-PL ([semi-structured interview; present and life-time version] in the German version; supplementary interview: Social behavior disorder, attention deficit and hyperactive disorder [14])

The time immediately preceding the start of therapy (two to four weeks) was defined as term t_1 _and further diagnostic instruments were implemented:

- LPC, Lifetime Parasuicide Count [15]

- THI, Treatment History Interview [16]

- GAF, Global Assessment Scale of Functioning [17]

- CGI, Clinical Global Impression [18]

- ILC, Inventory of Life Quality in Children and Adolescents [19]

- SCL-90-R, Symptom-Checklist-90-Revised [20]

- CBCL und YSR, Child Behavior Checklist und Youth-Self-Report [21,22]

- DIKJ, Depression Inventory for Children and Adolescents [23]

The point of time four weeks after end of the therapy program was defined as term t_2_. The same instruments as in term t_1 _were applied. The results of the therapy program four weeks after its end have been published elsewhere [11].

**Figure 1:**
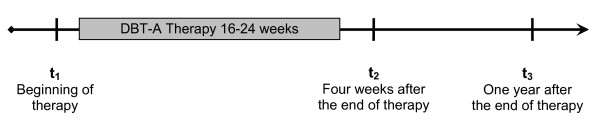
**Review of the investigation process of the Dialectical Behavioral Therapy for Adolescents (DBT-A)**.

At term t_3 _- one year after the end of therapy - we implemented the same instruments as in t_1 _(see Figure 1). We also followed up the instruments applied prior to admission. The study was approved by the review boards of the University of Freiburg. Written informed consent was obtained from all patients and their parents while children and adolescents gave their assent.

### Statistics

For statistical analysis, all patients who had started the therapy program were included in the data set (intent-to-treat analysis).

Changes occurring prior to therapy (t_1_), four weeks after therapy (t_2_) and one year after therapy (t_3_) were outlined as effect size (d) and p-levels of the Wilcoxon signed rank test.

Effect size was calculated according to the following formulae:

$$\text{Effect size }(d_{1}2)=\frac{\text{mean value }(t_{1})-\text{mean value }(t_{2})}{\sqrt{stddev{\left(t_{1}\right)}^{2}+stddev{\left(t_{2}\right)}^{2}}}$$

$$\text{Effect size }(d_{1}3)=\frac{\text{mean value }(t_{1})-\text{mean value }(t_{3})}{\sqrt{stddev{\left(t_{1}\right)}^{2}+stddev{\left(t_{3}\right)}^{2}}}$$

Mean values (t_1_, t_2, _t_3_) stand for the arithmetic mean value of the parameter value, while stddev's (t_1_, t_2, _t_3_) signify the standard deviation of the investigated variable at a particular time (t_1_= at the beginning of therapy, t_2 _= four weeks after therapy and t_3 _one year after therapy). Two-tailed p-values from Wilcoxon signed rank test were used for explorative data analysis.

## Results

### Changes in current psychiatric diagnoses and DSM-IV-Criteria for Borderline Personality Disorder (BPD)

Assessment at the beginning of therapy revealed that any patient had three, respectively four, current psychiatric DSM-IV axis-I diagnoses. Three adolescents showed two psychiatric DSM-IV axis-I diagnoses while two patients showed one. Five patients could not be diagnosed with any current psychiatric DSM-IV axis-I diagnoses.

At the beginning of therapy, each patient averaged 1.3 current psychiatric DSM-IV axis-I diagnoses (stddev 1.4, range 0 to 4 current psychiatric diagnoses per patient).

One year after the end of therapy, seven out of twelve adolescents could not be diagnosed with any current psychiatric DSM-IV axis-I diagnoses. At that time, four patients showed two psychiatric DSM-IV axis-I diagnoses while one patient showed one (see Table 1).

**Table 1:** Review of current psychiatric diagnoses on Axis I before (t1) and one year after therapy (t3) in the pilot study of Dialectical Behavioral Therapy for Adolescents (DBT-A)

Diagnosis of Axis I		**Number of current psychiatric diagnoses at the start of therapy (t**_**1**_**)**	**Number of current psychiatric diagnoses one year after therapy (t**_**3**_**)**
**F1X**	Harmful use and dependence syndrome of psychoactive substances (alcohol, cannabinoids and hallucinogens)	1	1
**F3X**	Affective disorders	4	2
**F4X**	Neurotic, stress-related and somatoform disorders	7	3
**F50**	Eating disorders	2	2
**F9X**	Behavioral and emotional disorders	1	1

One year after the end of therapy, each patient averaged 0.8 current psychiatric DSM-IV axis-I diagnoses (stddev 1.0, range 0 to 2 current psychiatric diagnoses per patient).

At the beginning of therapy, two patients (16%) fulfilled eight of the nine diagnostic criteria for BPD while one patient (8%) met seven, four patients (25%) six, three patients (8%) five and two patients (33%) four criteria.

All in all, a diagnosis for BPD according to DSM-IV was made for ten patients (83%) as they fulfilled five or more DSM-IV criteria.

From the beginning of therapy to one year after its end, the number of diagnostic criteria decreased distinctly. The mean value decreased from 5.8 (stddev 1.3), as of prior to therapy, to 2.75 (stddev 1.9) as of one year after therapy (effect size d = 0.78, p-level of Wilcoxon test = 0.003).

One year after the end of therapy, seven out of the nine diagnostic criteria for BPD were met by one patient (8%), five criteria were fulfilled by one patient (8%), four criteria by one patient (8%), three criteria by three patients (25%), two criteria by three patients (25%) and one criterion was met by two patients (17%) while one patient did not meet any diagnostic BPD criteria (8%) (see Table 2).

**Table 2:** Review of diagnostic criteria of borderline personality disorder before (t1) and one year after therapy (t3) in the pilot study of Dialectical Behavioral Therapy for Adolescents (DBT-A)

Diagnostic DSM-IV Criteria of borderline personality disorder	**Number of adolescents satisfying this criterion at the start of therapy (t**_**1**_**)**	**Number of adolescents satisfying this criterion one year after therapy (t**_**3**_**)**
Frantic efforts to avoid real or imagined abandonment	9	3
A pattern of unstable and intense interpersonal relationships characterized by alternating between extremes of idealization and devaluation	9	1
Identity disturbance: markedly and persistently unstable self-image or sense of self	8	2
Impulsivity in at least two areas that are potentially self-damaging (e. g. spending, sex, substance abuse, reckless driving and binge eating)	8	2
Recurrent suicidal behavior, gestures, threats or self-mutilating behavior	12	5
Affective instability due to a marked reactivity of mood (e. g. intense episodic dysphoria, irritability, or anxiety usually lasting a few hours and only rarely for more than a few days)	12	10
Chronic feelings of emptiness	8	3
Inappropriate, intense anger or difficulty in controlling anger (e. g. frequent displays of temper, constant anger and recurrent physical fights)	4	5
Transient, stress-related paranoid ideation or severe dissociative symptoms)	0	2

One year after the end of therapy, the diagnosis of BPD persisted in as few as two adolescents.

### Suicidal attempts, non-suicidal self-injurious behavior and inpatient treatments

The number and type of suicidal attempts and non-suicidal self-injurious behavior was investigated by using Lifetime Parasuicide Count (LPC) [15]. Before the start of therapy, 8 of 12 patients (67%) had attempted suicide at least once. Out of these, one patient had four suicide attempts, another had three suicide attempts while one patient had attempted suicide twice.

In the investigation group, suicidal attempts did neither occur during the treatment with DBT-A, nor in the year following therapy. All adolescents had shown non-suicidal self-injurious behavior and cutting of the skin of the forearms (mainly superficial) prior to therapy. In the month before admission to the study, non-suicidal self-injurious behavior had occurred in nine patients (75%). During this month, we registered an average of 4.3 (stddev 6.3) non-suicidal self-injurious behaviors per patient. During therapy, initial non-suicidal self-injurious behavior stopped quickly; however, it reoccurred in some patients at the end of therapy, which we take as being associated with disengaging from the therapist. In the month following therapy, eight patients (67%) showed no non-suicidal self-injurious behavior whereas four patients (33%) did; revealing a significant reduction of the target variable of DBT-A (effect size d = 0.89, p-level of Wilcoxon signed rank test = 0.018). One year after the end of therapy, seven patients (58%) still showed self-injurious behavior. Out of these, non-suicidal self-injurious behavior occurred once in one patient, twice in three patients, three times in one patient while one patient injured himself six times and another patient eleven times. In the year following the end of therapy, the number of non-suicidal self-injurious behaviors was significantly lower as compared with the month prior to therapy (effect size d = 0.92, p-level of Wilcoxon signed rank test = 0.015). There were no significant differences regarding non-suicidal self-injurious behavior between the end of therapy and the one-year follow-up.

Six adolescents (50%) had inpatient treatment at least once before admission to the study. During the year preceding therapy, each patient underwent on average 54 days of inpatient treatment. There was no need for inpatient treatment during therapy as well as up to four weeks after therapy. In the year following therapy, three of 12 patients (25%) had psychiatric inpatient treatment, whereby two of these dropped out of the DBT-A therapy.

### Therapy dropout

Nine out of twelve patients who started the program ended therapy regularly (75%). Two patients already stopped therapy after four and ten weeks, respectively. The first patient due to a strong reduction in self-injurious behavior after having completed the first skills section. The second one because therapy was considered as not being appropriate for him, owing to severe bulimic symptoms that required specific treatment. A third patient was not able to keep the appointments regularly due to extensive social phobic pathology. These three patients showed a very heterogeneous pattern both at the time four weeks after the scheduled end of therapy (t_2_) and at the time one year after therapy (t_3_). Of these patients, one showed an obvious amelioration of symptoms. A slight improvement of symptoms was found in the second patient, whereas psychosocial adjustment as well as psychopathology worsened in the third patient. The latter was the only patient requiring long-term inpatient treatment after participation in our study.

The results of the 12 adolescents included in the program are presented in the following.

### Comparison between psychosocial adjustment and quality of life prior to the start of therapy (t1) and one year after its end (t3)

Both the evaluation of overall functioning by using the Global Assessment Scale of Functioning (GAF) and the evaluation of global clinical impression by means of the Clinical Global Impression (CGI) showed significant amelioration under therapy, persisting one year after the end of therapy (effect size d (t_1_-t_3_) = -1.91, p-level of Wilcoxon signed rank test (t_1_-t_3_) = 0.010).

The CGI improved on average from "patient is markedly ill" to "patient is mildly ill" from prior to the start of therapy (t_1_) to one year after its end (effect size d (t_1_-t_3_) = 3.40, p-level in Wilcoxon signed rank test (t_1_-t_3_) = 0.007). Furthermore, a significant change in the global clinical impression occurred in the year following the end of therapy - not as distinct as during therapy though (effect size d (t_2_-t_3_) = 1.00, p-level of Wilcoxon signed rank test (t_2_-t_3_) = 0.011).

The average need for treatment, as detected by the Clinical Global Impression (CGI), went down from "outpatient treatment clearly necessary" to "outpatient treatment makes sense but is not absolutely necessary" over the course of therapy (d = 1.54; p = 0.007). This effect increased from prior to therapy to one year after its end (effect size d (t_1_-t_3_) = 2.20, p-level of Wilcoxon signed rank test (t_1_-t_3_) = 0.004).

The quality of life was self-evaluated by using the ILC adolescent (patient) version. The adolescent patients stated significant amelioration one month after the end of therapy regarding the following aspects: School (effect size d = 1.44; p = 0.026), interests and recreational activities (d = 0.79; p = 0.026), mental health (d = 1.65; p = 0.003), global rating of quality of life (d = 3.45; p = 0.002), stress associated with the present disorder (d = 1.58; p = 0.007) as well as stress associated with assessment and therapy (d = 1.60; p = 0.009). Regarding aspects such as family, social contact with peers and physical health, a tendency towards amelioration was documented which, however, did not reach any level of significance (see Table 3).

**Table 3:** Development of psychosocial adjustment from the beginning of therapy to one year after its end

Instrument			**Before therapy (t**_**1**_**)**		Four weeks after therapy (t_2_)		**One year after therapy (t**_**3**_**)**		**Statistics (t**_**1 **_**to t**_**3**_**)**	
		**N**	**Mean Value**	**SD**	**Mean Value**	**SD**	**Mean Value**	**SD**	**Wilcoxon-Test**	**Effect size d**
**GAF**	Overall functioning	12	57.8	12.0	76.7	8.7	78.3	9.4	p = 0.010**	-1.91
**CGI**	Clinical global impression	12	5.67	0.78	3.44	0.73	3.00	1.48	p = 0.007**	3.40
**ILC Adolescent**	School	9	3.00	1.78	1.86	1.07	1.80	1.03	p = 0.011*	1.85
	Family	11	2.92	1.24	2.13	1.25	2.00	0.78	p = 0.070	0.79
	Social contact with peers	11	2.17	0.72	1.63	0.52	1.73	0.79	p = 0.206	0.62
	Interests and recreational activities	11	2.50	1.17	1.38	1.06	1.82	1.25	p = 0.107	0.46
	Physical health	11	2.50	1.17	2.25	0.89	2.27	0.91	p = 0.366	0.18
	Mental health	11	3.83	0.72	2.50	1.20	2.36	0.67	p = 0.004**	2.05
	Global rating of life quality	11	3.67	0.58	1.88	0.83	1.91	0.54	p = 0.004**	2.84
	Stress associated with the present disorder	11	3.83	0.94	2.50	1.07	2.18	0.87	p = 0.009**	1.77
	Stress associated with the assessment and therapy	11	2.25	0.87	1.13	0.35	1.27	0.47	p = 0.026*	1.30
**ILC Therapist**	Social contact with peers	12	2.17	1.19	1.78	1.30	1.83	1.34	p = 0.102	0.27
	Self occupation	12	2.17	0.58	2.44	2.51	1.58	0.67	p = 0.020*	0.94
	Need for treatment	12	4.08	0.29	2.67	0.71	2.25	1.14	p = 0.004**	2.20

SD = Standard deviation; * = Significance (bilateral) ≤ 0.05; ** = Significance (bilateral) ≤ 0.01; GAF = Global Assessment Scale of Functioning; CGI = Clinical Global Impression; ILC = Inventory of Life Quality in Children and Adolescents

One year after the end of therapy, its effect persisted in each of the mentioned aspects except for interests and recreational activities:

School (effect size d (t_1_-t_3_) = 1.85; p (t_1_-t_3_) = 0.011, Mental health (d (t_1_-t_3_) = 2.05; p = 0.004), Global rating of life quality (d (t_1_-t_3_) = 2.84; p (t_1_-t_3_) = 0.004), Stress associated with the present disorder (d (t_1_-t_3_) = 1.77; p (t_1_-t_3_) = 0.009) and Stress associated with assessment and therapy (d (t_1_-t_3_) = 1.30; p (t_1_-t_3_) = 0.026).

### Comparison between psychopathology prior to the start of therapy (t1) and one year after its end (t3) by means of self-evaluation

Psychopathology was measured by means of the Symptom-Checklist SCL-90-R [20], the Youth Self Report of the Child Behavior Checklist (YSR) [21,22] and the Depression Inventory for Children and Adolescents (DIKJ) [23].

The SCL-90-R provided proof of a significant amelioration within the time period between the start of therapy (t_1_) and one year after its end (t_3_). The Global Severity Index (effect size d (t_1_-t_3_) = 1.30; p-level of Wilcoxon-test (t_1_-t_3_) = 0.008), the Positive Symptom Distress Index (d (t_1_-t_3_) = 1.08; p (t_1_-t_3_) = 0.016) and the Positive Symptom Total (d (t_1_-t_3_) = 1.27; p (t_1_-t_3_) = 0.013) showed a reduction of psychopathology.

The adolescents' self-reported symptoms according to the SCL-90-R decreased significantly between the start of therapy (t_1_) and one year after therapy (t_3_) as shown in the following Primary Symptom Dimensions: Depression (d(t_1_-t_3_) = 2.14; p(t_1_-t_3_) = 0.004), Anxiety (d(t_1_-t_3_) = 1.05; p(t_1_-t_3_) = 0.014), Somatization (d(t_1_-t_3_) = 0.68, p(t_1_-t_3_) = 0.028) and Interpersonal Sensitivity (d(t_1_-t_3_) = 1.49, p(t_1_-t_3_) = 0.011).

Two other Primary Symptom Dimensions showed significant changes between the start of therapy (t_1_) and the time point of one month after therapy (t_2_): Obsessive-Compulsive (d = 1.82; p = 0.025) and Hostility (d = 0.95; p = 0.013). One year after the end of therapy, no significant reduction in self-reported symptoms within these dimensions could be found any longer.

Regarding the remaining dimensions Phobic Anxiety, Paranoid Ideation and Psychoticism, there were no significant changes. This might possibly be due to having hardly registered any symptoms in the beginning of therapy; especially in the dimensions Phobic Anxiety, Paranoid Ideation and Psychoticism.

Similar to the SCL 90-R, the YSR showed significant amelioration in all global indices regarding the time between the start of therapy and one year after therapy. The global score (d (t_1_-t_3_) = 1.82; p(t_1_-t_3_) = 0.003) as well as the broad-band scales Internalising (d(t_1_-t_3_) = 1.54; p(t_1_-t_3_) = 0.007) and Externalising Behavior (d (t_1_-t_3_) = 0.57; p(t_1_-t_3_) = 0.008) showed a reduction in psychopathology.

In the following subscales of the YSR, psychopathological symptoms decreased significantly from the start of therapy to one year after therapy:

Social Withdrawal (d(t_1_-t_3_) = 1.13; p(t_1_-t_3_) = 0.016), Anxious/Depressed (d(t_1_-t_3_) = 1.47; p(t_1_-t_3_) = 0.008), Schizoid-Obsessive (d(t_1_-t_3_) = 1.04; p(t_1_-t_3_) = 0.011), Attention Problems (d(t_1_-t_3_) = 1.46; p(t_1_-t_3_) = 0.005) and Aggressive Behaviors (d(t_1_-t_3_) = 0.54; p(t_1_-t_3_) = 0.014).

In the subtests Somatic Complaints, Social Problems and Delinquent Behaviors, we observed a pronounced - yet not significant - tendency towards an ameliorated self-evaluation from the start of therapy to one year after therapy (see Table 4).

**Table 4:** Development of psychopathology from the beginning of therapy to one year after its end

Instrument			**Before therapy (t**_**1**_**)**		**Four weeks after therapy (t**_**2**_**)**		**One year after therapy (t**_**3**_**)**		**Statistics (t**_**1 **_**to t**_**3**_**)**	
		N	Mean Value	SD	Mean Value	SD	Mean Value	SD	Wilcoxon-Test	Effect size d
**SCL-90 R**	Global Severity Index	11	0.93	0.38	0.57	0.21	0.44	0.33	p = 0.008**	1.30
	Positive Symptom Total	11	44.4	12.9	36.3	10.4	27.1	16.5	p = 0.016*	1.08
	Positive Symptom Distress Index	11	1.84	0.39	1.4	0.19	1.37	0.29	p = 0.013*	1.27
	G1 Somatization	11	0.65	0.43	0.5	0.49	0.36	0.28	p = 0.028*	0.68
	G2 Obsessive-Compulsive	11	0.99	0.55	0.6	0.38	0.6	0.4	p = 0.052	0.70
	G3 Interpersonal Sensitivity	11	1.21	0.51	0.92	0.49	0.46	0.44	p = 0.011*	1.49
	G4 Depression	11	1.63	0.6	0.79	0.47	0.48	0.4	p = 0.004**	2.14
	G5 Anxiety	11	0.84	0.39	0.4	0.26	0.35	0.47	p = 0.014*	1.05
	G6 Hostility	11	1.08	0.59	0.57	0.45	0.85	0.73	p = 0.563	0.24
	G7 Phobic Anxiety	11	0.43	0.61	0.25	0.42	0.29	0.5	p = 0.572	0.23
	G8 Paranoid Ideation	11	0.54	0.32	0.52	0.37	0.32	0.32	p = 0.233	0.58
	G9 Psychoticism	11	0.42	0.4	0.3	0.21	0.18	0.22	p = 0.075	0.64
	S10 Additional Items	11	9.42	5.04	5.88	2.85	4.91	3.65	p = 0.032*	1.01
**YSR**	Global Score	11	71.5	11.3	45.5	22.6	41.8	19.2	p = 0.003**	1.82
	Internalizing Behavior	11	26.8	5.3	15.9	8.1	14.6	9.3	p = 0.007**	1.54
	Externalizing Behavior	11	19.7	8.4	15.2	9.5	14.5	7.4	p = 0.008**	0.57
	Social Withdrawal	11	7.08	2.61	4.38	3.16	3.91	2.77	p = 0.016*	1.13
	Somatic Complaints	11	4.0	2.63	2.63	2.13	3.09	2.94	p = 0.505	0.26
	Anxious/Depressed	11	17.08	4.5	9.75	5.2	8.09	7.03	p = 0.008**	1.47
	Social Problems	11	4.0	2.22	2.13	1.64	1.91	2.21	p = 0.058	0.86
	Schizoid/Obsessive	11	3.0	2.34	1.5	1.51	0.91	1.45	p = 0.011*	1.04
	Attention Problems	11	8.67	2.35	3.88	2.03	4.45	3.14	p = 0.005**	1.46
	Delinquent Behaviors	11	6.42	3.26	5.0	2.62	4.55	2.07	p = 0.041*	0.54
	Aggressive Behaviors	11	13.25	5.71	10.13	7.02	9.91	5.74	p = 0.014*	0.54
**DIKJ**	Average Score Per Item	10	0.78	0.2	0.45	0.21	0.41	0.23	p = 0.022*	1.51

SD = Standard deviation; * = Significance (bilateral) ≤ 0.05; ** = Significance (bilateral) ≤ 0.01; SCL-90-R = Symptom Checklist 90 revised; YSR = Youth Self Report of the Child Behavior Checklist; DIKJ = Depression Inventory for Children and Adolescents

The DIKJ (Depression Inventory for Children and Adolescents), a self-evaluation instrument for depressive symptoms, showed significant improvements as well. One year after therapy, the patients estimated their depressive psychopathology to be significantly lower than at the beginning of therapy (d(t_1_-t_3_) = 1.51; p(t_1_-t_3_) = 0.022)._._

Furthermore, changes during therapy were evaluated by parents. Unfortunately, it was not possible to analyze these data as it remained incomplete, owing to difficult biosocial environments; e. g., no contact with father or mother, respectively lack of parents' compliance.

## Discussion

The DBT-A, as evaluated in this study, is based upon a manual which has been translated and modified for use in Germany by our study group. Thus, the results of this study represent the first experiences gained with DBT-A in German-speaking countries. Our study aimed at investigating whether suicidal and non-suicidal self-injurious behavior decreased in the treated adolescents, whether the adolescents completed the therapy program successfully and whether psychosocial adjustment and psychopathology of patients improved and consistently remained this way over a one-year period up to follow-up.

Adolescents with suicidal and non-suicidal self-injurious behavior and traits of a borderline personality disorder are considered to be a patient group which is difficult to treat. Therefore, the therapy drop-out rate in this patient group is known to exceed 60% [24]. The fact that a therapy program which takes place twice a week and stretches across 16 to 24 weeks is apparently able to bring about positive changes in behavior, psychosocial adjustment and in the distress associated with the adolescents' symptoms, is especially motivating. Furthermore, the majority of patients are generally able to complete therapy regularly.

By using this therapy, our investigation group was able to show a stable reduction of suicidal and non-suicidal self-injurious behavior over the course of one year - as considered being the primary target of DBT. Our results validate evaluations from the US, which were able to prove a reduction of suicidal and non-suicidal self-injurious behavior under the treatment with DBT in comparison to controls. This applies to both female adults and adolescents diagnosed with BPD symptoms [6,7,25]. In a 10-year prospective follow-up study on adult patients with BPD by Zanarini et al. [26], 50% of patients recovered from borderline personality disorder which was defined as a remission of symptoms as well as social and vocational functioning during the previous two years. It has to be emphasized that certain symptoms of BPD, e. g. non-suicidal self-injury, suicide gestures and suicide attempts, are easier to remediate with medication, psychotherapy or a combination of both [27]. Furthermore, a 1-year open trial by Goldstein et al. could demonstrate a significant improvement in suicidality and non-suicidal self-injurious behavior in adolescents with bipolar disorder [9]. However, these very promising results on the efficacy of DBT are challenged to some extent as Linehan's biosocial theory on BPD - suggesting that individuals with BPD have biologically based abnormalities in emotion regulation contributing to more intense and rapid responses to emotional stimuli (invalidation in particular) - has not fully been proved yet [28]. Woodberry et al. have found neither self-report nor physiological evidence of any hyperarousal in BPD groups [28].

The second important goal in the hierarchy of DBT is to keep patients in therapy. In our study, the drop-out rate amounted to 25%, which ranks slightly below the drop-out rate of 38% as found in a comparable study by Rathus and Miller [7,9]. Taken together, with completion rates between 62% and 90%, this corresponds with the current literature on DBT [7,9]. Our drop-out rate still ranks far below Rathus' and Miller's control group's drop-out rate of 60%, which underwent unspecific `treatment as usual`. Remarkably, the patients treated with DBT had a higher impact of psychiatric diagnoses before the start of therapy than the control group [7].

In accordance to comparable studies [6,7,25], our patient group exhibited a reduction of the length of psychiatric inpatient treatment during therapy.

After therapy, patients appear to be dealing with the various and sensitive demands of adolescent evolution more easily. This hypothesis is also based on the improvement of both the Global Level of Functioning and the reduction of the need for treatment as assessed by the therapist.

Patients dropping out of therapy showed more current psychiatric DSM-IV axis-I diagnoses at the beginning of therapy (i. e. on average 1.3 diagnoses per patient), rather than the patients who ended therapy regularly (i. e. 0.9 diagnoses per patient). This tendency increased one year after the end of therapy. At that time, a total of nine current psychiatric DSM-IV axis-I diagnoses were assessed. Out of these, six diagnoses (67%) occurred in the three patients having dropped out of therapy while the nine patients ending therapy regularly were diagnosed with merely three diagnoses (33%).

At the beginning of therapy, the diagnosis of BPD was assessed for 83% of the adolescent patients, whereas one year after the end of therapy, this diagnosis persisted in only 17% of patients. Out of the nine patients ending therapy regularly, only one patient was still suffering from BPD according to the diagnostic criteria of DSM-IV. one year after therapy. This corresponds to a remission of BPD one year after therapy in six out of seven patients (86%) who ended therapy regularly. In comparison, Zanarini et al. [26,29] have stated similar remission rates under different kinds of therapy (35% after two, 49% after four, 69% after six years and 93% after 10 years) in a 10-year follow-up study on adult patients suffering from BPD.

The distinct reduction of suicidal and non-suicidal self-injurious behavior during therapy is reflected in the rating of the DSM-IV borderline criteria assigned to these symptoms. The adolescents made clear progress in the DSM-IV criteria "unstable and intense interpersonal relationships", "identity disturbance" and "impulsivity". These criteria were explicitly discussed in the multi family skills training group and solution strategies were developed in the training modules Distress Tolerance Skills and Emotion Regulation Skills. The adolescents' significant improvements are in line with the improved scores on SCL-90-R Interpersonal Sensitivity and Depression subscales. Distinct progress occurred in the DSM-IV criterion "frantic efforts to avoid real or imagined abandonment", indicating that patients generally improve in getting along with themselves and their environment and have more self-confidence after the end of therapy. Patients dropping out of therapy met more DSM-IV criteria per patient when starting therapy than patients who ended therapy regularly. During the observed period, there was less reduction of fulfilled DSM-IV criteria per patient in those patients who dropped out of therapy.

The number of fulfilled DSM-IV criteria for BPD per patient as well as the number of current psychiatric DSM-IV axis-I diagnoses before the start of therapy could thus provide a predictive statement as to whether a particular patient will be able to pass through therapy completely, and as to how far the implementation of therapy will make sense.

Under therapy, self-evaluation (SCL 90-R, YSR, DIKJ) in particular showed improvements in the global scores of psychopathology, persistent over the year following therapy. In self-evaluation, the symptoms of depression (SCL 90-R, YSR, DIKJ), anxiety (SCL 90-R, YSR), social withdrawal (YSR) and attention problems (YSR) decreased in particular. Rathus and Miller [7] have found similar results in SCL 90-R. In addition, they have assessed an improvement of social contacts. In our study, this effect kept limited to the year following therapy.

The adolescents' quality of life, measured by using ILC, improved clearly from the start of therapy to one year after therapy.

Assessment by the parents showed an improvement of the quality of life, both during therapy, and in the year following therapy. Symptoms of psychopathology in general diminished - mostly in the year after therapy.

All in all, the three patients who dropped out of therapy presented an amelioration regarding their situation prior to therapy. In one patient, the symptoms vanished quickly. Pathology improved so much after having passed the first skills section, that the adolescent and his family abandoned further treatment. One year after therapy, one patient showed slightly reduced pathology. In one patient, pathology persisted undiminished after therapy dropout. The influence of incomplete participation on the development of patients remains unclear.

Limitations of the present study are mainly related to its design. The study lacks a control group by means of which the strong therapeutic effects over the course of therapy could be compared to controls. The fact that the reliability and validity of the diagnosis of BPD in adolescents as well as its measurements have not been evaluated sactisfactorily yet, limits the present study results to some extent. As assessments were conducted by therapists, a potential bias cannot be ruled out.

## Conclusions

Our pilot study aimed at establishing DBT-A in German-speaking countries to survey its practicability and to provide first results on the effectiveness of the treatment. On the basis of our promising findings, we consider this treatment program worth further evaluation. Thus, the conceptuation for a multicentre, randomized, controlled study, which compares DBT-A to conventional outpatient psychotherapy is required.

## Competing interests

The authors declare that they have no competing interests.

## Authors' contributions

ES participated in the design of the study. CF conceived of the study and performed the statistical analyses. CF, RB, BS, CB and CS participated in the execution of the study and carried out the therapy. All authors reviewed and approved the manuscript.
